# Basic and clinical research, and the review of original papers

**DOI:** 10.1590/s1980-57642008dn10200001

**Published:** 2007

**Authors:** Ricardo Nitrini

**Affiliations:** Editor-in-Chief

It is certainly intriguing to observe how little we know about the research that has been carried out in Brazil, even in our particular field of interest. While clinical neuroscientists may have a reasonable knowledge of the scientific production of their fellow clinicians, they are frequently unaware of the contributions by basic neuroscientists; and vice-versa. The main reason for this may lie in the lack of adequate dissemination of our scientific output. Even papers that have been published in journals with high impact factor in each specific area rarely break the virtual barrier that exists between different areas of the same field. This phenomenon almost certainly occurs in other developing countries, and to justify it by assuming that researchers only know their specific area does not fully explain the issue, since everyone knows the eminent international basic and clinical researchers as well as the papers they publish in their fields of expertise. In this case, the virtual barrier is probably broken by the very high impact factor of the journals and through astute use of the increased opportunity for diffusion that the authors and their colleagues have open to them.

This second issue of **Dementia & Neuropsychologia**, besides reviews and original manuscripts on clinical research, also contains papers from basic research, making clear that the scientific community recognizes the importance of this propagation, not only toward due acknowledgment in their own country, which is doubtless important, but primarily because it is necessary in establishing and multiplying cooperative studies. The link between basic and clinical researchers is also one of the key objectives of the organizers of the forthcoming Meeting of Researchers on Alzheimer Disease and Related Disorders, to be held in Ouro Preto, December 6 to 8, 2007, which hopefully will represent a milestone in our scientific production in this field.

**Dementia & Neuropsychologia** has been receiving original manuscripts from many Brazilian centers, demonstrating the confidence held in our Editorial Board, and we wish to acknowledge this essential support and to reciprocate with the diffusion of the papers, and future indexation of the journal. In order to continue to receive new original manuscripts it is felt fitting that the editorial principles of **Dementia & Neuropsychologia** be made even more transparent. When receiving a manuscript our reviewers are provide with the framework partially reproduced below:

1. Consider every manuscript as a candidate for publication – As **Dementia & Neuropsychologia** is not yet indexed, it is possible that a reasonable proportion of the submitted papers will not yet be of high scientific quality before the peer-review process.
2. Manuscripts can be improved by the peer-review process – A good reviewer should indicate the strengths and weaknesses of the manuscript, and may mention how the study could have been designed to reach a firmer conclusion, but should also try to give instruction on how to improve the quality of the manuscript using the available data, or data that are easily accessible by the author(s). Orientation on re-writing each section of the manuscript may be included. Guidance on rearranging the statistical analyses is very important and should be incorporated when required.
3. Stimulate authors to advance in their research careers and to submit further manuscripts to **Dementia & Neuropsychologia** – Irony or acidic criticisms should be avoided. Reviewers should write “as a knowledgeable, courteous colleague advising the author(s)”.^1^ This is particularly important for countries, or for areas of study, where there is no solid scientific tradition.
4. Avoid vague proclamations; cite the appropriate papers – For example, if there are other papers that deserve citation in the text, the reviewer should cite them. If the manuscript is not novel, the work that it replicates should be cited.^1^
5. Every part of the manuscript, from title to conclusion(s) may warrant and receive attention – The title, abstract and conclusions of a manuscript are extremely important. A good title constitutes the smallest number of words that adequately defines the content of a paper.^2^ As is often said, few people will read the whole paper, but many will read the title, and if they eventually reach the abstract, they will go on to the conclusions. The conclusion(s) should be clearly supported by the data.
6. Comment on spelling and grammar only if they compromise the scientific accuracy of the manuscript – A final review of the use of the English language will be available for every accepted manuscript.
7. Finally, one of the main roles of this journal is to bring to light papers of high scientific quality that are not well known either because they have been published in Portuguese, Spanish or other languages, or because they focus on Third World populations. The reviewers should urge the authors to cite such papers, when appropriate.

**Ricardo Nitrini** Editor-in-Chief
